# Why do they do it? A grounded theory study of the use of low-value care among primary health care physicians

**DOI:** 10.1186/s13012-020-01052-5

**Published:** 2020-10-21

**Authors:** Sara Ingvarsson, Hanna Augustsson, Henna Hasson, Per Nilsen, Ulrica von Thiele Schwarz, Mia von Knorring

**Affiliations:** 1 Procome Research Group, Medical Management Centre, Department of Learning, Informatics, Management and Ethics, Karolinska Institutet, SE-171 77 Stockholm, Sweden; 2 Unit for Implementation and Evaluation, Center for Epidemiology and Community Medicine (CES), SE-171 29 Stockholm, Stockholm Region Sweden; 3 Department of Health, Medical and Caring Sciences, Linköping University, Linköping, Sweden; 4 School of Health, Care and Social Welfare, Mälardalen University, Box 883, 721 23 Västerås, Sweden; 5 Leadership in Healthcare and Academia Research Group, Medical Management Centre, Department of Learning, Informatics, Management and Ethics, Karolinska Institutet, SE-171 77 Stockholm, Sweden

**Keywords:** De-implementation, Low-value care, Primary health care, Physicians, Lab tests, Grounded theory, Sweden

## Abstract

**Background:**

The use of low-value care (LVC) is widespread and has an impact on both the use of resources and the quality of care. However, few studies have thus far studied the factors influencing the use of LVC from the perspective of the practitioners themselves. The aim of this study is to understand why physicians within primary care use LVC.

**Methods:**

Six primary health care centers in the Stockholm Region were purposively selected. Focus group discussions were conducted with physicians (*n* = 31) working in the centers. The discussions were coded inductively using a grounded theory approach.

**Results:**

Three main reasons for performing LVC were identified. *Uncertainty and disagreement about what not to do* was related to being unaware of the LVC status of a practice, guidelines perceived as conflicting, guidelines perceived to be irrelevant for the target patient population, or a lack of trust in the guidelines. *Perceived pressure from others* concerned patient pressure, pressure from other physicians, or pressure from the health care system. *A desire to do something for the patients* was associated with the fact that the visit in itself prompts action, symptoms to relieve, or that patients' emotions need to be reassured. The three reasons are interdependent. Uncertainty and disagreement about what not to do have made it more difficult to handle the pressure from others and to refrain from doing something for the patients. The pressure from others and the desire to do something for the patients enhanced the uncertainty and disagreement about what not to do. Furthermore, the pressure from others influenced the desire to do something for the patients.

**Conclusions:**

Three reasons work together to explain primary care physicians’ use of LVC: uncertainty and disagreement about what not to do, perceived pressure from others, and the desire to do something for the patients. The reasons may, in turn, be influenced by the health care system, but the decision nevertheless seemed to be up to the individual physician. The findings suggest that the de-implementation of LVC needs to address the three reasons from a systems perspective.

Contributions to the literature

- Low-value care (LVC) research hitherto does not provide insights into *why* health care professionals use LVC.
- Previous research has focused on factors related to the individual professionals (e.g., physicians), thus neglecting the potential relevance of other factors, including system-level factors, for LVC use.
- This study identifies three interdependent reasons that explain primary care physicians’ use of LVC
- De-implementation of LVC needs to address multiple reasons from a systems perspective.

## Background

Health systems worldwide are required to provide high-quality health care for the population. At the same time, there are growing demands for accountability and effective use of resources [1]. To achieve these goals, health systems are increasingly expected to both adopt evidence-based practices and de-implement (reduce or discontinue) practices that are not supported by research evidence, usually referred to as low-value care (LVC) [2]. LVC has been defined as practices lacking clinical effectiveness (and therefore not being cost-effective), which have a poor risk-benefit profile or which are not supported by adequate evidence [1]. Examples of LVC range from overuse of clinical tests to overtreatment and overdiagnosis [3]. The most common type of LVC is the inappropriate use of an otherwise effective practice for patients for whom the benefit has not been demonstrated [4]. Low-value care thus includes both practices that benefit no one and practices that are beneficial to a selected patient category.

The use of LVC is common. US data show that 14–16% of all care provided to patients is of low value [5]. In recent years in Sweden, there are also indications of increased practices that should not be used according to the Swedish guidelines [6]. Thus, LVC poses a significant problem in relation to the quality of care and cost-effectiveness.

Different strategies have been used to decrease the use of LVC. Publishing guidelines on what practices to avoid is one attempt to influence the practitioners. Choosing Wisely guidelines have been published in many countries to reduce the use of specific LVCs [7]. However, merely listing practices to avoid does not solve the LVC problem [8]. Other types of strategies that have been studied include clinical decision support [9], provider feedback [10], education for clinicians [11] and patients [12], and financial incentives [13]. In their systematic review, Colla et al. [14] found that the most common strategy was clinical decision support and showed that there is considerable evidence for the effectiveness of clinical decision support and provider feedback. However, the strongest support was found for multicomponent strategies that addressed both patients and providers. The review highlighted that most strategies have not been based on an analysis of factors influencing the use of LVC (i.e., determinants for LVC) and that there is a lack of knowledge on how to engage clinicians in the de-implementation process. Thus, in order to better understand how different strategies can be effective, there is a need for studies that identify determinants for the use of LVC and that take the perspective of clinicians.

Most of the prior studies on determinants for the use of LVC have focused on identifying what factors impact LVC use rather than *why* different factors have an impact. For example, the following factors have been found to be positively associated with receiving LVC: age and gender of the patient (e.g., [15, 16]) and experience or specialty of the physician (e.g., [17]). Findings have been contradictory, which makes it difficult to draw a firm conclusion about the determinants. Few studies have asked health care professionals about the determinants for their use of LVC. One exception was a nationwide survey of US physicians that found the most common reason was a concern for malpractice, followed by the willingness to be safe in the decision and the need for more information to reassure themselves [18].

LVC research hitherto does not provide insights into *why* health care professionals use LVC. Furthermore, previous studies have mainly focused on factors related to individual professionals (e.g., physicians), thus neglecting how individual- and system-level factors might interact to influence the use of LVC. To obtain a fuller understanding of the influences on the use of LVC, more in-depth analysis is required. To that end, this study intends to utilize a grounded theory approach to explore why physicians within primary care use LVC.

## Methods

We collected data through six semi-structured focus group discussions (FGDs) with a total of 31 physicians subjected to qualitative analysis. We chose a qualitative approach because there is limited knowledge concerning why primary care physicians use LVC, and we considered FGDs the most relevant method for gaining a deeper understanding of this issue.

### Study setting

We conducted the study within public primary health care in Stockholm Region, which has the largest population of the 21 regions in Sweden. Swedish primary health care is part of the tax-funded health care system, which is governed by the regions [19]. Extensive discussions were held with managers within different parts of health care in order to identify examples of LVC. Primary care was found to be a good setting for discussing LVC. Three lab tests, defined by experts on LVC, were used as examples and as a basis for the recruitment of centers: erythrocyte sedimentation rate (ESR), vitamin D test, and aspartate transaminase (AST) [20–22]. The criteria for selection specified that the examples of LVC were well defined, all experts agreed that it was LVC, and statistics were available that show a difference in use between settings. The lab tests were chosen to obtain as large variation as possible with regard to how LVCs are prescribed.

### Recruitment of participants

We invited all 66 public primary health care centers by e-mail to participate in the study. Seventeen centers accepted the invitation. Then, we compared these centers in terms of their use of the three lab tests previously defined as LVC.

We used a purposeful sampling method. Out of the17 centers, we included in this study the centers with the highest and the lowest prescribing rate of one of these three lab tests in order to capture as rich information as possible and to get a large variation of settings concerning their use of LVC. The chosen centers were low/high prescribers in comparison with all 66 centers. We estimated that six centers would be sufficient to explore the purpose of the study [23]. In the next stage, we asked each center’s manager to invite all physicians at the six centers (ranging from three to 25 physicians per center) to participate in the study. A total of 31 accepted and were invited to FGDs at their center together with their colleagues. The FGDs included three to seven participants. We selected physicians because they are the professional group responsible for prescribing lab tests in Swedish primary health care.

### Data collection

We chose FGDs as the data collection method in order to capture the shared experience from the members of the center as well as each physician’s individual perspective [24]. We used the same interview guide for all FGDs. We constructed the interview guide to capture both physicians’ thoughts about LVC in general and the three low-value lab tests in particular. We used the chosen lab tests in the FGDs as practical examples to discuss the use of LVC practices. Then, we encouraged participants to freely share their thoughts on LVC and how and why different factors (individual and organizational) impacted the use of and the de-implementation of LVC practices. We also informed participants that we were interested in their perspectives on LVC and what, in their view, constitutes LVC. We emphasized that there were no right or wrong answers and that their way of working would not be criticized. Themes covered in the interview guide included the participants’ general view on low-value care; what they perceived as helpful and unhelpful when trying to avoid LVC; individual, group, and organizational strategies to reduce the use of LVC; and their perspective on the three lab tests.

The first author (SI) led all FGDs, acted as a facilitator for the discussions, and explained the purpose of the discussion and aspects of confidentiality. A research assistant took the role of an observer. Participants were informed that participation was voluntary and that they had the right to withdraw from the study at any time. Informed consent was collected before the FGD. Each FGD lasted approximately 45 min and was audio-recorded and transcribed verbatim.

### Data analysis

We analyzed the data using a grounded theory approach as described by Corbin [25]. The transcribed FGDs were read several times in order to get a sense of the overall content. In the first step, the first author identified meaning units and coded them through a process of line-by-line coding in Microsoft Word and then grouped the codes into preliminary categories and subcategories in Microsoft Excel. Parallel to this process, memos were written to capture preliminary ideas and thoughts around the meaning of the data. In the next step, the first author further expounded on these categories and memos together with the last author. Then, the authors tested these ideas by returning to the material and validating or discarding the results. Throughout the analysis process, all authors discussed and validated the findings. In the next step, a conceptual model was formulated to illustrate the relationship between the categories.

The last author is an expert in qualitative analysis. All authors have considerable experience with research and development projects within health care and are well familiar with primary care and the work situation of physicians. The team is multiprofessional and multidisciplinary.

In the “Results” section, we will illustrate the categories and subcategories through rich quotes from the FGDs using “[]” to show when a text has been omitted and “()” when a text has been added. We made changes in the quotes for practical reasons but did not alter the meaning of the statements.

## Results

The grounded theory analysis revealed three main reasons (i.e., categories) for why physicians use LVC. Each category consisted of three or more subcategories. We describe the categories and subcategories in the following and present a conceptual model that illustrates the relationship between the reasons.

### Uncertainty and disagreement about what not to do

In the analysis, we identified several aspects (subcategories) leading to situations when the physicians felt they were questioning what they should not do.

#### Being unaware of the LVC status

Simply not being aware of a practice regarded as LVC was one aspect that made it difficult for physicians to know what to do and what not to do. The process of keeping updated on the latest guidelines for all different patient categories was perceived as challenging. The physicians described a complex process that included reading articles, discussing with colleagues, listening to experts, and subscribing to newsletters. One of the participants described it like this:

*Every time I participate in education, I think to myself, this is completely new knowledge to me. It is a scary experience since we are expected to be updated on so many different topics. (IP2, FGD1).*

They claimed that it was almost impossible to keep updated on new information and to know when they had the correct information or not. One participant described it as the following:

*But we do so incredibly many things, so how do you know – out of the thousand million things I do – what is a habit and what is something that I ought to question? It is absolutely impossible! (IP3, FGD1).*

#### Guidelines perceived to be conflicting

Guidelines and routines exist at different levels of the health care system (e.g., national, regional, and at the center), and these sometimes contradict each other. For example, one of the lab tests defined as LVC–AST was included in a local clinical guideline, sending the message that it should be done. The ordering system for lab tests furthered this problem with preset local standards. One participant formulated it this way:

*We get such mixed messages from different sources (IP15, FGD3).*

#### Guidelines perceived to be irrelevant for the patient population

Guidelines could be difficult to interpret and were sometimes not regarded as relevant for the patient group within primary care. One participant described it like this:

*But the problem is that the guidelines are relevant for a fairly small portion of our patients. (IP27, FGD5).*

The patient population within primary health care was viewed as different from the populations in specialized care because this patient group often presented symptoms that could be related to a variety of diagnoses that make it difficult to follow guidelines for each possible scenario. One participant described it like this:

*I believe that many of those writing the guidelines are organ specialists and work based on a selected patient sample, and then they expect us to do the same within primary care (IP13, FGD3).*

Moreover, some guidelines recommending a low use of a specific practice were considered irrelevant for a certain patient population. One example was the prevalence of tuberculosis in the uptake area of the health care center, which, according to the participants, could warrant a higher level of prescriptions of ESR.

*Spontaneously, it is difficult to compare, because we need to prescribe vitamin D and ESR tests more often because a lot of our patients have tuberculosis, or it is more common, and other inflammatory parasite-related illnesses, and we have many more who have a vitamin-D deficiency (IP 8, FGD2).*

#### Lack of trust in the source of the guidelines

For some of the guidelines, the physicians did not trust the source of the recommendation or guideline. Physicians sometimes knew which expert had been involved in constructing a specific guideline. They expressed doubts about the correctness of the guidelines or personal anecdotes on the process behind the construction of the guidelines, furthering the difficulty in following a specific guideline. One participant described the process behind the guidelines with regard to how personal conflicts had influenced the instructions in the guideline recommendations.

*But this (guideline) is some sort of compromise in order to avoid people getting angry. (IP6, FGD1)*

The participants even talked about the development of guidelines being driven by individual physicians’ agendas. One participant expressed it this way:

*I feel that the discussion about AST and ALT that has been promoted nationally by a (specific) physician is fairly uninteresting (IP 2, FGD1).*

### Perceived pressure from others

The second reason, perceived pressure from others, had three sources (subcategories).

#### Patient pressure

Two types of scenarios were described by the physicians. Some patients expressed clear expectations for a specific practice, and some expressed expectations in receiving any kind of procedure. This was often related to their previous experiences of successful practices or based on information that the patients gathered before the visit. One participant provided this example:

*It is the expectations from the patients. Many of them act as if they are ordering some sort of merchandise. (IP29, FGD6).*

Not receiving the requested practice was not always accepted and could result in unsatisfied patients, which was something the physicians wanted to avoid. Not accommodating the patient’s request could furthermore lead to the patient seeking care from another physician for the same problem. One of the participants described it in this way:

*Every time, I diagnose the patient based on my competence and experience, but the patient is not satisfied and wishes to take lab tests. It is always much easier to accommodate the patient and order those tests or examinations (IP 21, FGD 4).*

#### Pressure from other physicians

Specialist physicians from outside the center sometimes requested certain tests as a criterion for accepting a referral for a patient. Not complying with the request implied not being able to refer the patient to the correct care provider. One participant described it this way:

*Most of the time they (specialists) do not accept the patient before we have ordered the lab tests and the examinations that they requested (IP8, FGD2).*

Requests from other physicians also involved patients being referred to the center with a request for a physician to prescribe LVC. Physicians described this as easier to comply with than going against the other physician and doing their own assessment of the need for the specific practice. One participant described it this way:

*It can be problematic when new, inexperienced physicians within the emergency department order us to perform unnecessary tests. Some people may not critically evaluate the order and simply comply with the request (IP31, FGD6).*

#### Pressure from the health care system to perform unnecessary tasks

The physicians also mentioned multiple other practices that they perceived as LVC. Those were not listed as LVC in any clinical guidelines but were perceived as LVC since they cost time and money without any clear benefits. Examples of those practices included the following: patient visits for non-severe symptoms or rushed visits where patients were perceived as being able to wait longer before seeing a physician. Administrative tasks included the registration of patient visits in different digital systems in order to get the right financial compensation and routine follow-up visits for certain patient groups. Further, unnecessary visits yield more situations where the physicians can be influenced by pressure from the patients and a desire to do something for the patients.

*So that is what I feel perhaps is the most low value that we do (meeting) healthy people who should not be here that actually cost the most money (IP27, FGD5).*

The demands from the health care management entailed financial incentives for performed interventions and written directives asking for a certain amount of a specific intervention. Not complying with the demands from the management could result in less financial support for the center.

*It is not cheating the system – it is exactly the way it is designed to work. We simply have to shake hands with more people this year than last year (IP 6, FGD1).*

One example they described was the opportunity for patients to schedule their appointment themselves via the Internet. Those visits were often perceived as unnecessary and driving unnecessary practices.

*The more often you see a physician, the more likely you are to get a lab work, an X-ray, to get a treatment (IP3, FGD1).*

The pressure from patients, other physicians, and the health care system was further enhanced through the lack of counter pressure from the system to not prescribe LVC. One of the participants described it this way:

*So many factors influence if we are updated or not, know our job or not, and if we do not get any support or help mistakes can happen. There is no control system. (IP3, FGD1).*

### A desire to do *something* for the patients

Besides demands from others, a wish to do something for each patient was also a reason for providing LVC. This category was different from pressure from others since the participants described situations where no specific requests were made by the patients, but the physician still wanted to do something for the patient. Three aspects (subcategories) leading up to this desire were identified.

#### The visit in itself prompts action

The scheduled visit with a patient could by itself be a reason for prescribing LVC. Participants described that providing a practice was part of the process. They also described that it was easier to refrain from prescribing a LVC if there was an alternative practice they could prescribe instead. One participant described it like this:

*...and that is the problem: if you are to remove a habit, it is always nice to do something (IP1, FGD1).*

#### Symptoms need to be relieved

Another aspect of this was the symptoms described by the patients. Some of the symptoms could be harmless to the patient but still experienced as painful; this created a challenge for the physician when he or she could not help reduce these symptoms. Sympathy for the patients’ symptoms was part of the reason for wanting to do something for the patients. One participant described it this way:

*It is enough to have had a bad cough after having had a cold yourself, trying desperately with a cough medication, and finally being able to fall asleep to feel sympathy for those who need it (IP 2, FGD1).*

Some symptoms would have a preferred but perhaps unavailable intervention. One of the participants described it this way:

*Moderate depressions, for example, where you prescribe antidepressants, instead of scheduling follow-up visits or when there are no available psychologist appointments (IP13, FGD 3).*

#### Patients’ emotions need to be reassured

Physicians described that not only the symptoms but also the emotions of the patients influenced the use of LVC. Anxious patients were difficult to calm without doing something despite the fact that they did not request any specific intervention. The physicians felt that the only way to help the patients with their worry would be to order some tests. One of the participants described it this way:

*It is easier to take a couple of tests so that the patient will let go of their worry and be reassured that everything is all right (IP9, FGD2).*

### Why do they do it?—interdependent reasons that combined explain the use of LVC

**Fig. 1:**
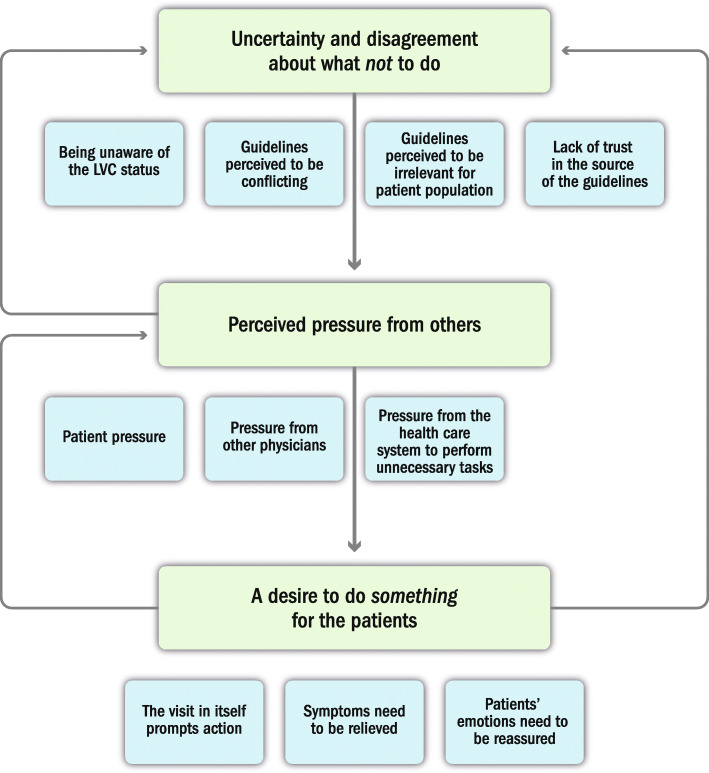
Why do they do it? Interdependent reasons that combined explain the use of LVC

The analysis showed that the three reasons described above both independently and combined can explain why physicians use LVC (Fig. 1). Uncertainty and disagreement about what not to do make physicians vulnerable to pressure from others to provide the practice (e.g., a treatment) and more likely to give in to the desire to do *something* for the patients*.* The perceived pressure from others can also influence physicians’ interpretation of guidelines, thus making them more uncertain as to whether a practice really should be considered LVC. Similarly, the desire to do something for the patient could also make physicians more likely to agree to the perceived pressure from others.

*It is easier when you have something to back it up when patients come and express a desire to get something, (saying) ‘but I got it from the previous (doctor)’. If there are clear guidelines, you can say no (IP4, FGD1).*

Avoiding the use of LVC was perceived as challenging, with no simple solutions for how to achieve it. The task is made even more complex due to influences emerging from different parts of the health care system, such as requests from other physicians and management. However, despite these multilevel influences on LVC use, the physicians reported that they felt left alone to manage the situation.

## Discussion

The aim of this study was to explore the reasons physicians use LVC. The results showed that physicians generally had a broader definition of LVC and that the use was influenced by three interdependent reasons: uncertainty and disagreement on what *not* to do, perceived pressure from others, and a desire to do something for the patients. This finding underscores the importance of addressing more than one reason when attempting to reduce physicians’ LVC use.

The interaction of different factors is in line with determinant frameworks such as Consolidated Framework for Implementation Research (CFIR) and Integrated-Promoting Action on Research Implementation in Health Services (i-PARIHS) used within implementation science, which emphasize the interdependence of various factors [26]. However, in contrast to several determinant frameworks, the results of our study emphasized the strong impact of patient expectations on receiving LVC. It is noteworthy that determinant frameworks such as Theoretical Domains Framework and Active Implementation Frameworks do not explicitly account for patients as a potential determinant of successful implementation [26]. From an academic perspective, the definition of LVC may seem simple, as it is described in relation to research evidence, cost-effectiveness, and harm to patients or the public health [1, 27]. Many LVC practices are also described in guidelines that recommend for or against the use of a specific practice for a specific patient group (e.g., [28, 29]). However, viewing the concept of LVC from a practitioner’s perspective, it is considerably more complex than the straightforward scientific definition implies. The process of keeping updated on the latest guidelines is time-consuming, and there seems to be a lack of prompts for what guidelines to examine and when. As a reason, uncertainty and disagreement about what *not* to do resonate with previous research pointing to numerous barriers to guideline use, including conflicting recommendations in guidelines [30], non-applicable guidelines [31], and clinicians not being convinced of a practice being LVC [32]. This is in line with previous implementation research stating that knowledge is not enough to succeed with change [33].

Primary care is the first level of care that patients receive, which means that practitioners in this setting meet a broad range of patients with disparate problems [19]. The fact that many of the patients within primary care have multimorbid diagnoses makes adherence to specific, individual clinical guidelines even more complex [34]. The results in this study show that the heterogeneous patient population makes the amount of diagnoses to keep updated on larger than for any other specialties in the health care system. Furthermore, the results point to simply being aware of a guideline is not sufficient because there may be conflicting recommendations, problems in applying the guidelines to the specific patient or patient population, and problems in trusting the source of the guidelines. This result is in line with the conclusions of a research by Gabbay and Le May [35] that has shown that clinicians face a more complex reality than the recommendations in guidelines typically describe. Strategies to reduce uncertainty and disagreement regarding what not to do need to account for the complexity of the issue. This could include reminders about the guidelines that appear at the point of care and feedback to practitioners on their use of LVC. Furthermore, discussions on how to interpret the guidelines as well as feedback from the practitioners on perceived problems with the guidelines are also important to address uncertainty and lack of trust in guidelines.

Our study found that the physicians had a broader definition of LVC than previously described in the literature. Their perception of LVC also included organizational aspects such as patients being able to schedule their own visits via the Internet and time limits for waiting time, resulting in sometimes unnecessary visits. The physicians argued that some of the practices that management requested were not relevant, and they therefore considered them to be LVC. This reasoning is in line with the argument by von Thiele Schwarz et al. [36] emphasizing that health care has multiple values to consider and that different stakeholders may perceive values differently. One such example was visits perceived as unnecessary that led to increasing other LVC because physicians responded to patients’ inappropriate requests in order to feel that they had done something for the patients. Studies on unnecessary visits have mainly focused on emergency department visits of patients with non-acute problems [37, 38]. Reorganization with more available primary care does not seem to lessen the problem but could release a latent need for physician visits as health care becomes more available [39]. The role of physicians as gatekeepers to the community’s common resources has been questioned before [40], but in this study, the results can be interpreted as a wish from the physicians to focus the health care resources to those with the most urgent need.

We found that perceived pressure from patients and other physicians influenced the physicians’ use of LVC. This has been identified in previous research (e.g., [41]). Acting on the information has been described as “a bias towards action” [42] and therefore something that is difficult to refrain from. It has been suggested that it may be easier for physicians to avoid low-value practices when they can offer alternatives [43]. However, Zikmund-Fisher et al. suggested that a bigger concern than the prevalence of patient requests is that the physicians perceive the requests as a barrier for reducing LVC [44]. Tannenbaum and colleagues [45] found that patients who were informed about the risks with a LVC were less likely to demand a LVC, and teaching physicians specific communication strategies seems to be helpful in reducing the use of LVC [46].

The developed model shows how the different reasons interact. Physicians’ reasons for using LVC are influenced by the entire health care system, but the physicians have to work with reducing LVC on their own. This suggests that strategies aimed at de-implementing low-value practices could benefit from a systems perspective. Such an implication is consistent with the findings of Colla et al. [14] that showed multicomponent strategies were more effective than single-component interventions for de-implementing LVC and why publishing guidelines is not enough [8]. Future research should focus on system factors that influence LVC and what strategies on a system level can be successful in reducing the use of LVC. Furthermore, more knowledge is needed on what mechanisms within multicomponent strategies influence the results and what strategies can reduce different types of LVC.

### Methodological considerations

Some caution must be taken when interpreting the results of this study. The study was performed in primary care in Stockholm Region. Stockholm is a capital city in an urban region. A free choice model is applied, implying that all citizens can choose their primary care provider (both private and public) regardless of where they live. This implies that even though Sweden has a publicly funded, universal health care system, there is still competition between primary care centers which may increase the pressure from patients. Thus, multiple aspects in the region and the organization of care may differ from other primary care settings and thus impact the transferability of the findings to other settings. The choice to use lab tests (e.g., LVC) to achieve maximum variation in LVC practice could have resulted in findings relevant only for lab tests. However, the lab tests were presented at the end of the FGDs, which rather deepened the discussions on the reasons for using LVC. The described reasons for using the lab tests were similar to the reasons related to other examples of LVC.

There are several factors influencing the credibility of the study. The focus group format facilitated access to the physicians’ discussions and a multifaceted picture of the reasons for LVC use. Using the physicians’ regular weekly meetings allowed access to participants that may not have been possible to reach through other means. The data suggest that six FGDs produced rich data and that the number of centers included was sufficient to saturate the categories and subcategories. Studies also suggest that six focus groups would most likely be enough to obtain rich information about an issue [23]. The grounded theory analysis made it possible to identify not only a list of reasons but also how they interacted and the overall complexity of the LVC issue.

## Conclusions

Three reasons work together to explain primary care physicians’ use of LVC: uncertainty and disagreement about what not to do, perceived pressure from others, and the desire to do something for the patients. The reasons may, in turn, be influenced by the health care system, but the decision nevertheless seemed to be up to the individual physician. The findings suggest that the de-implementation of LVC must address the three reasons from a systems perspective.

## Data Availability

The datasets used will be available from the corresponding author on reasonable request.

## Abbreviations

FGD: Focus group discussion
LVC: Low-value care

## References

[1] Garner S, Littlejohns P. Disinvestment from low value clinical interventions: NICEly done? BMJ. 2011;343 Available from: https://www.bmj.com/content/343/bmj.d4519. 10.1136/bmj.d4519 21795239

[2] Foy R, Sales A, Wensing M, Aarons GA, Flottorp S, Kent B (2015). Implementation science: a reappraisal of our journal mission and scope. Implement Sci.

[3] Morgan DJ, Dhruva SS, Coon ER, Wright SM, Korenstein D (2018). 2017 update on medical overuse: a systematic review. JAMA Intern Med.

[4] McWilliams JM, Schwartz AL (2017). Focusing on high-cost patients—the key to addressing high costs?. N Engl J Med.

[5] Niven DJ, Mrklas KJ, Holodinsky JK, Straus SE, Hemmelgarn BR, Jeffs LP (2015). Towards understanding the de-adoption of low-value clinical practices: a scoping review. BMC Med.

[6] Lång väg till patientnytta – en uppföljning av nationella riktlinjers inverkan på vården i ett decentraliserat system. 2015.

[7] Levinson W, Kallewaard M, Bhatia RS, Wolfson D, Shortt S, Kerr EA (2015). ‘Choosing Wisely’: a growing international campaign. BMJ Qual Saf.

[8] Rosenberg A, Agiro A, Gottlieb M, Barron J, Brady P, Liu Y (2015). Early trends among seven recommendations from the choosing wisely campaign. JAMA Intern Med.

[9] Goldzweig CL, Orshansky G, Paige NM, Miake-Lye IM, Beroes JM, Ewing BA (2015). Electronic health record-based interventions for improving appropriate diagnostic imaging: a systematic review and meta-analysis. Ann Intern Med.

[10] Hogli JU, Garcia BH, Skjold F, Skogen V, Smabrekke L. An audit and feedback intervention study increased adherence to antibiotic prescribing guidelines at a Norwegian hospital. BMC Infect Dis. 2016;16(96) Available from: http://ovidsp.ovid.com/ovidweb.cgi?T=JS&CSC=Y&NEWS=N&PAGE=fulltext&D=med8&AN=26920549. 10.1186/s12879-016-1426-1 PMC4769530 26920549

[11] Yesudian GT, Gilchrist F, Bebb K, Albadri S, Aspinall A, Swales K (2015). A multicentre, multicycle audit of the prescribing practices of three paediatric dental departments in the North of England. Br Dent J.

[12] Gonzales R, Corbett KK, Wong S, Glazner JE, Deas A, Leeman-Castillo B (2008). “Get Smart Colorado”: impact of a mass media campaign to improve community antibiotic use. Med Care.

[13] Menya D, Platt A, Manji I, Sang E, Wafula R, Ren J, et al. Using pay for performance incentives (P4P) to improve management of suspected malaria fevers in rural Kenya: a cluster randomized controlled trial. BMC Med. 2015;13(1) Available from: http://www.embase.com/search/results?subaction=viewrecord&from=export&id=L606522722. 10.1186/s12916-015-0497-y PMC4608124 26472130

[14] Colla CH, Mainor AJ, Hargreaves C, Sequist T, Morden N (2017). Interventions aimed at reducing use of low-value health services: a systematic review. Med Care Res Rev.

[15] Lalude OO, Gutarra MF, Pollono EN, Lee S, Tarwater PM (2014). Inappropriate utilization of SPECT myocardial perfusion imaging on the USA-Mexico border. J Nucl Cardiol.

[16] Singh A, Bodukam V, Saigal K, Bahl J, Wang Y, Hanlon A, et al. Identifying risk factors associated with inappropriate use of acid suppressive therapy at a community hospital. Gastroenterol Res Pract. 2016;7 Available from: %3CGo. 10.1155/2016/1973086 PMC5080516 27818680

[17] Silverman M, Povitz M, Sontrop JM, Li L, Richard L, Cejic S (2017). Antibiotic prescribing for nonbacterial acute upper respiratory infections in elderly persons. Ann Intern Med.

[18] Choosing Wisely. Unnecessary tests and procedures in the health care system. What physicians say about the problem, the causes, and the solutions: Choosing Wisely; 2014. Available from: http://www.choosingwisely.org/wp-content/uploads/2015/04/Final-Choosing-Wisely-Survey-Report.pdf. Cited 2019 Dec 9.

[19] The National Board of Health and Welfare. Primärvårdens uppdrag - En kartläggning av hur landstingens uppdrag till primärvården är formulerade. Stockholm; 2016.

[20] Brigden ML (1999). Clinical utility of the erythrocyte sedimentation rate. Am Fam Physician.

[21] Rockwell M, Kraak V, Hulver M, Epling J (2018). Clinical management of low vitamin D: a scoping review of physicians’ practices. Nutrients..

[22] Seppanen K, Kauppila T, Pitkala K, Kautiainen H, Puustinen R, Iivanainen A (2016). Altering a computerized laboratory test order form rationalizes ordering of laboratory tests in primary care physicians. Int J Med Inform.

[23] Guest G, Namey E, McKenna K (2017). How many focus groups are enough? Building an evidence base for nonprobability sample sizes. Field Methods.

[24] Wilkinson S, Silverman D (2016). Analysing focus group data. Qualitative research.

[25] Corbin JM (2015). Basics of qualitative research: techniques and procedures for developing grounded theory. 4th edition. Strauss AL, editor.

[26] Nilsen P (2015). Making sense of implementation theories, models and frameworks. Implement Sci.

[27] Brody H (2010). Medicine’s ethical responsibility for health care reform—the top five list. N Engl J Med.

[28] Irfan N, Brooks A, Mithoowani S, Celetti SJ, Main C, Mertz D (2015). A controlled quasi-experimental study of an educational intervention to reduce the unnecessary use of antimicrobials for asymptomatic bacteriuria. PLoS One.

[29] Christian J, VanHaaren A, Cameron K, Lapane K (2004). Alternatives for potentially inappropriate medications in the elderly population: treatment algorithms for use in the Fleetwood Phase III Study. Consult Pharm.

[30] Han PK, Klabunde CN, Noone AM, Earle CC, Ayanian JZ, Ganz PA (2013). Physicians’ beliefs about breast cancer surveillance testing are consistent with test overuse. Med Care.

[31] Bell HT, Steinsbekk A, Granas AG (2015). Factors influencing prescribing of fall-risk-increasing drugs to the elderly: a qualitative study. Scand J Prim Health Care.

[32] Kerns JW, Winter JD, Winter KM, Boyd T, Etz RS (2018). Primary care physician perspectives about antipsychotics and other medications for symptoms of dementia. J Am Board Fam Med.

[33] Grimshaw J, Eccles MP, Jonson E (2008). Knowledge translation of research findings.

[34] Boyd CM, Fortin M (2010). Future of multimorbidity research: how should understanding of multimorbidity inform health system design?. Public Health Rev.

[35] Gabbay J, Le May A (2011). Practice-based evidence for healthcare: clinical mindlines.

[36] von Thiele Schwarz U, Aarons GA, Hasson H (2019). The Value Equation: three complementary propositions for reconciling fidelity and adaptation in evidence-based practice implementation. BMC Health Serv Res.

[37] Breen BM, McCann M (2013). Healthcare providers attitudes and perceptions of “inappropriate attendance” in the emergency department. Int Emerg Nurs.

[38] Almoosa KF, Luther K, Resar R, Patel B (2016). Applying the new institute for healthcare improvement inpatient waste tool to identify “waste” in the intensive care unit. J Healthc Qual.

[39] Martin A, Martin C, Martin PB, Martin PA, Green G, Eldridge S (2002). “Inappropriate” attendance at an accident and emergency department by adults registered in local general practices: how is it related to their use of primary care?. J Health Serv Res Policy.

[40] Wammes JJ, Jeurissen PP, Verhoef LM, Assendelft WJ, Westert GP, Faber MJ. Is the role as gatekeeper still feasible? A survey among Dutch general practitioners. Fam Pract. 31(5):538–44 Available from: http://ovidsp.ovid.com/ovidweb.cgi?T=JS&CSC=Y&NEWS=N&PAGE=fulltext&D=med8&AN=25135953. 10.1093/fampra/cmu046 25135953

[41] Selby K, Cornuz J, Cohidon C, Gaspoz J-M, Senn N (2018). How do Swiss general practitioners agree with and report adhering to a top-five list of unnecessary tests and treatments? Results of a cross-sectional survey. Eur J Gen Pract.

[42] Ayanian JZ, Berwick DM (1991). Do physicians have a bias toward action? A classic study revisited. Medical Decision Making.

[43] Voorn VMA, de Mheen PJ, Wentink MM, Kaptein AA, Koopman-van Gemert AWMM, So-Osman C (2014). Perceived barriers among physicians for stopping non–cost-effective blood-saving measures in total hip and total knee arthroplasties. Transfusion..

[44] Zikmund-Fisher BJ, Kullgren JT, Fagerlin A, Klamerus ML, Bernstein SJ, Kerr EA (2017). Perceived barriers to implementing individual Choosing Wisely® recommendations in two national surveys of primary care providers. J Gen Intern Med.

[45] Tannenbaum C, Martin P, Tamblyn R, Benedetti A, Ahmed S. Reduction of inappropriate benzodiazepine prescriptions among older adults through direct patient education: the EMPOWER cluster randomized trial. JAMA Intern Med. 174(6):890–8 Available from: http://ovidsp.ovid.com/ovidweb.cgi?T=JS&CSC=Y&NEWS=N&PAGE=fulltext&D=med8&AN=24733354. 10.1001/jamainternmed.2014.949 24733354

[46] May L, Franks P, Jerant A, Fenton J. Watchful waiting strategy may reduce low-value diagnostic testing. J Am Board Fam Med JABFM. 29(6):710–7 Available from: http://ovidsp.ovid.com/ovidweb.cgi?T=JS&CSC=Y&NEWS=N&PAGE=fulltext&D=medl&AN=28076254. 10.3122/jabfm.2016.06.160056 28076254

